# Insights into Nuclear G-Protein-Coupled Receptors as Therapeutic Targets in Non-Communicable Diseases

**DOI:** 10.3390/ph14050439

**Published:** 2021-05-07

**Authors:** Salomé Gonçalves-Monteiro, Rita Ribeiro-Oliveira, Maria Sofia Vieira-Rocha, Martin Vojtek, Joana B. Sousa, Carmen Diniz

**Affiliations:** 1 Laboratory of Pharmacology, Department of Drug Sciences, Faculty of Pharmacy, University of Porto, 4050-313 Porto, Portugal; salomemonteiro8180@gmail.com (S.G.-M.); ritarmo23@gmail.com (R.R.-O.); msrocha@ff.up.pt (M.S.V.-R.); matovoj@gmail.com (M.V.); 2 LAQV/REQUIMTE, Faculty of Pharmacy, University of Porto, 4050-313 Porto, Portugal

**Keywords:** GPCRs, cancer, cardiovascular diseases, ligands, nuclear GPCR signaling, GPCR-based therapeutics

## Abstract

G-protein-coupled receptors (GPCRs) comprise a large protein superfamily divided into six classes, rhodopsin-like (A), secretin receptor family (B), metabotropic glutamate (C), fungal mating pheromone receptors (D), cyclic AMP receptors (E) and frizzled (F). Until recently, GPCRs signaling was thought to emanate exclusively from the plasma membrane as a response to extracellular stimuli but several studies have challenged this view demonstrating that GPCRs can be present in intracellular localizations, including in the nuclei. A renewed interest in GPCR receptors’ superfamily emerged and intensive research occurred over recent decades, particularly regarding class A GPCRs, but some class B and C have also been explored. Nuclear GPCRs proved to be functional and capable of triggering identical and/or distinct signaling pathways associated with their counterparts on the cell surface bringing new insights into the relevance of nuclear GPCRs and highlighting the nucleus as an autonomous signaling organelle (triggered by GPCRs). Nuclear GPCRs are involved in physiological (namely cell proliferation, transcription, angiogenesis and survival) and disease processes (cancer, cardiovascular diseases, etc.). In this review we summarize emerging evidence on nuclear GPCRs expression/function (with some nuclear GPCRs evidencing atypical/disruptive signaling pathways) in non-communicable disease, thus, bringing nuclear GPCRs as targets to the forefront of debate.

## 1. Introduction

G-protein-coupled receptors (GPCRs) comprise a large and versatile protein superfamily known to mediate responses to several extracellular stimuli namely small molecules, peptides and proteins, photons, etc., that regulate a broad spectrum of physiological functions, such as vision, smell and taste. GPCRs also modulate neurological, cardiovascular, endocrine and reproductive functions [1,2,3,4]. GPCRs regulatory actions have justified the past and current intensive research in the development of GPCR ligands and their applicability and use in different economic sectors of society.

Based on the knowledge gathered so far, GPCR ligands are being used to generate products with commercial value for diverse types of industries such as pharmaceutical, food and cosmetic. Some of these products take advantage of GPCRs’ properties as cell communication modulators in physiological processes involving the sensorial system. Examples of that are the taste and smell [5,6,7]. Accordingly, some GPCR ligands can be incorporated into products to ameliorate their sensorial properties and, consequently, improve their acceptability by consumers. Furthermore, some studies also indicate that certain GPCRs can mediate sweet-taste synergisms (for instance, sweeteners, neohesperidin dihydrochalcone and cyclamate), which can potentiate the response of cells to sucrose, [8] providing a way to reduce sugar content in food. Taking advantage of this knowledge, several studies are currently undergoing with the view to develop sugar substitutes for a healthy diet, which has a major impact on human life and well-being. This subject has been the focus of intense research in the last few years and is particularly important for patients with metabolic diseases.

Another group of GPCR ligands has been designed with the goal to treat or prevent diseases that involve, for instance, the cardiovascular, nervous or endocrine systems. In this regard, GPCRs are targets for nearly 35% of the drugs currently in use in clinical practice [9], and comprise more than 100 types of receptors [10] divided into six classes according to the following classification [11,12]: rhodopsin-like (A), secretin receptor family (B), metabotropic glutamate (C), fungal mating pheromone receptors (D), cyclic adenosine monophosphate (cAMP) receptors (E) and frizzled (F), where classes D and E are not found in vertebrates.

Ligands can act to promote GPCRs activation (agonists) or, by opposition, to block the activity exerted by GPCRs (neutral antagonists or inverse agonists). Currently, ligands for GPCR mainly belong to rhodopsin-like receptors (94%), and from these, the aminergic receptors constitute the group with more receptor types. These ligands include antagonists (that block the receptors‘ activity induced by endogenous ligands, namely hormones, neurotransmitters, among others), but also agonists (full, partial and inverse agonists) which are able to activate and modulate GPCR responses, being used for specific therapeutic applications. Intense research focusing on the development of new GPCR-based therapeutics, using for example crystallography, biosensors [13,14,15] or computational chemistry [16] have allowed the appearance of a new generation of drugs, some of them revisiting older targets, but others are looking for ligands that do not bind directly to the ligand binding pocket. Examples are inverse agonists, allosteric modulators and drugs targeting GPCR heterodimers. As a consequence, an increasing number of patents on GPCR ligands are currently under evaluation concerning its value/safety in clinical trials involving several types of diseases (please see as an example [17]). During the past decade, drug repositioning studies have extended the therapeutic indications of many drugs currently in use in clinical practice including drugs that bind to GPCRs [1,18,19].

Activation of plasma membrane GPCRs usually is mediated by the binding of an agonist that stabilizes the active conformation of the receptor with the subsequent recruitment and activation of intracellular signaling pathways [20]. Indeed, when GPCRs are activated, receptors may couple to several heterotrimeric G proteins, namely Gα_i/o_, Gα_q/11_, Gα_12/13_ and G_βγ,_ to regulatory proteins such as G protein-coupled receptor kinases (GRKs) and β-arrestins, and then activate downstream signaling pathways [21,22]. Moreover, dimerization activation, transactivation, biased activation, biphasic activation, and intracellular activation of GPCR have also been recently described as forms of activation of GPCRs, increasing the complexity of the GPCR activation processes (for more information on this subject, please see [2,23]).

Classically, GPCRs are considered as cell surface receptors with seven-transmembrane domains localized in the plasma membrane but scientific data revealed that a large number of GPCRs are also present in intracellular sites, including in the nuclei (e.g., in the nuclear membrane), the nuclear GPCRs. These receptors seem to have an important biological impact and gained interest due to their putative role in the regulation of gene transcription, both in physiology and in disease, namely in non-communicable diseases. In this review we focus on the most recent findings related to nuclear GPCRs expression and function demonstrating the biological relevance of GPCRs localized in the nuclear membrane, particularly in non-communicable diseases, where atypical and disruptive signaling events have been reported, thus highlighting nuclear GPCRs as new therapeutic targets to the forefront of debate. In addition, GPCR ligands and the development of strategies to selectively discriminate the newly identified nuclear GPCRs will also be discussed.

## 2. Nuclear GPCRs in Physiological Conditions

In 1971, Robertson and Kijairallah provided the first sign indicative of a new localization for GPCRs [24], by injecting angiotensin II (Ang II) in a nuclear zone and observing ultrastructural changes. Consequently, a renewed interest had led to an intensive research in this field and to a shift from a dogmatic view that considered GPCRs as functional receptors exclusively present in the plasma membrane, to a view that appoints for the existence of GPCRs in intracellular compartments [1,25,26,27]. Indeed, in initial studies, the intracellular localization of GPCRs was interpreted as a way of receptor traffic within the cell (from the nuclei to membrane or vice versa) but findings showing an intracellular localization of GPCRs [28] that have a functional role [1,25] have extended the way GPCRs are now viewed.

Nuclear GPCRs seem to have a widespread distribution in the organism, and reports have shown their occurrence particularly in the cardiovascular and nervous system, although their presence has also been described in the nuclear membrane of cells from other anatomical regions such as liver, kidney, cornea and bones [27,29]. The presence of GPCRs in the nuclear membrane has been established experimentally in culture cells (Human embryonic kidney (HEK-293), Mouse melanoma (B16) and Chinese hamster ovary (CHO) cells, cardiomyocytes, vascular smooth muscle cells, neurons, among others type of cells) but also in tissue cells from animal models (heart, hypothalamus, thalamus, septum, midbrain, liver, kidney and spleen [27]). In these studies, the nuclear localization of GPCRs has been identified both in isolated nuclei or intact cells using radioligand binding assays, electron and/or confocal microscopy techniques and western blot [30]. In addition to morphological studies, the actions exerted by nuclear GPCRs have been assayed using methods that enable to segment the nuclear GPCRs’ activation (relatively to respective plasma membrane counterparts), involving, for instance, cytosolic microinjections [30], caged and/or photolyzed ligands [31].

It has been established that GPCRs can be found in the nuclear envelope, at the inner and/or outer membranes, positioned in a way that allows the effector-binding domain to face the cytosol or the nucleoplasm [32]. The way GPCRs appear in the nuclear membrane seems to occur in many ways, as previously reviewed [27,33,34]. Briefly, GPCRs might be synthesized in the endoplasmic reticulum or within the nucleus, and then traffic to the nuclear membrane constituting nuclear GPCRs. Another possible source for nuclear GPCRs is the internalization of plasma membrane GPCRs and their subsequent translocation into the nuclear membrane via an agonist-dependent pathway [1]. Interestingly, some reports also indicate that GPCRs translocation from plasma to nuclear membrane can also occur upon activation via an agonist independent pathway (please see [27]). Moreover, short peptide sequences of basic amino acid residues (usually lysine/glycine-arginine repeats) present in the C terminus or in the intracellular loop of GPCRs, have been identified as nuclear localization sequences (NLS) [35] and these sequences appear to be involved in the translocation of GPCRs to the nucleus. NLS motif seems to orientate GPCRs to the nucleus through importin mechanisms and/or small GTPases [34,36]. On the other hand, the translocation process can also be independent of the NLS motif, with some receptors such as bradykinin type 2 (B2) (although presenting a NLS sequence) requiring heterodimerization with lamin C [37] to translocate to the nuclear membrane. In addition, some other sequences of peptides (different from the classical NLS) can also foment the nuclear import of receptors as observed with the 38 amino acid long fragment of heterogeneous nuclear ribonucleoproteins A1 and A2 proteins (the M9 sequence) that can be recognized by transportin [27].

It is important to emphasize that the GPCRs that have been identified in the nuclear membrane mostly belong to class A GPCRs, from where 22 receptor families and 34 GPCR subtypes have been described in a nuclear localization. In addition, some receptors belonging to class B (two families and three subtypes), class C (two families and three subtypes) and class F (one family and one subtype) have also been identified in the nuclear membrane [27].

The way nuclear GPCRs can be activated still needs further studies in order to clarify if its behavior is aligned with that described for plasma membrane GPCRs. Nevertheless, studies conducted so far indicate that the classical activation mediated by ligands and subsequent activation of signaling pathways described for plasma membrane GPCRs seems to also occur in nuclear GPCRs. Indeed, innumerable studies support that in physiological conditions nuclear GPCRs activation (and subsequent events) occurs within the nucleus by pathways involving second messengers, ion channels, proteins, etc., that are commonly associated with plasma membrane GPCRs [1,30,38,39]. In this sense, GPCRs localized in the nuclear membrane can trigger and modulate nuclear calcium levels, cAMP generation, nitric oxide (NO) and/or reactive oxygen (ROS) and/or nitrogen species (RNS) production, which, in turn, will condition the regulation of gene transcription and, thus, of multiple physiological processes, namely of cell proliferation, transcription, apoptosis, angiogenesis and survival [1,31,37]. Overall, these data highlight the nucleus as an autonomous signaling organelle containing functional nuclear GPCRs.

## 3. Nuclear GPCRs Activation: Endogenous and Exogenous Ligands

**Figure 1:**
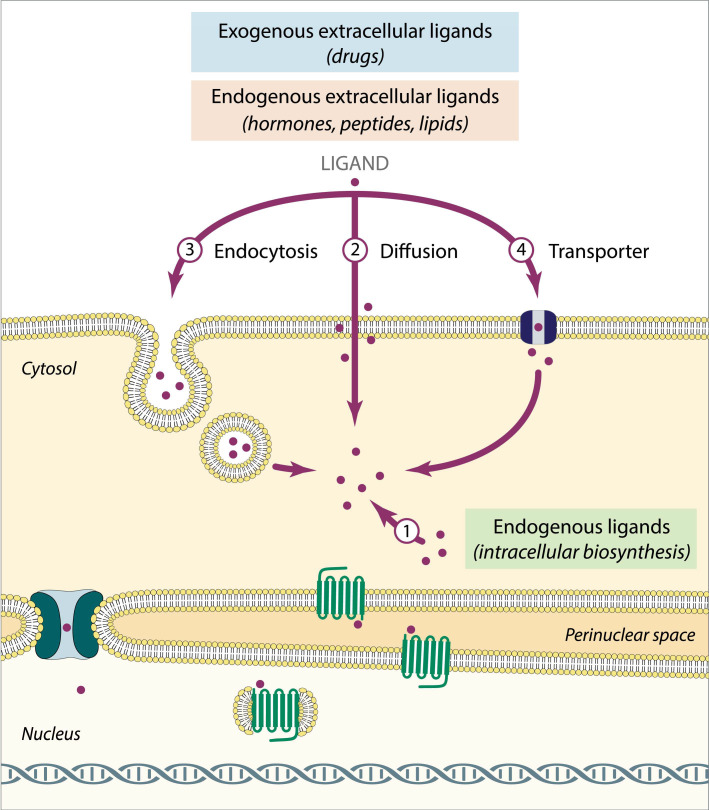
Activation of nuclear G-protein-coupled receptors (GPCRs). Exogenous and endogenous (extracellular and intracellularly produced): (1) biosynthesis of endogenous ligands inside the cell; (2) diffusion of extracellular ligands; (3) endocytosis of extracellular ligands; (4) active transport of extracellular ligands (transporters and exchangers).

The complete knowledge regarding the way nuclear GPCRs are activated still needs further studies but a large amount of data describes that activation of nuclear GPCRs results from the interaction of the receptor with exogenous or endogenous ligands. Endogenous ligands are synthesized in the organism, in other cell types or within the cell itself (Figure 1). The later process appears to occur intracellularly and increases the ligand bioavailability near the nucleus in order to activate nuclear GPCRs. As such, the intracellularly produced ligands seem to constitute the preferential candidates to bind to nuclear GPCRs. Examples of that type of ligands are apelin, bradykinin, Ang II [40] and prostanoids [41] including prostaglandins, platelet-activating factor lysophosphatidic acid [42] and endothelin [43]. For another type of ligands, namely extracellular ligands (both exogenous and endogenous), their bioavailability to interact with nuclear GPCRs depends largely on their respective ability to cross the plasma membrane and, thus, access nuclear GPCRs: ligands that are lipophilic molecules may freely trespass the plasma membrane and reach the nucleus (by diffusion), whereas for ligands with less plasma membrane permeability (with more hydrophilic properties), its uptake requires alternative ways such as endocytosis or transport through membrane exchangers and/or transporters (for example: glutamate [40], urotensin-II [44], etc.).

Despite efforts regarding the development of new GPCR ligands in the last decades, few studies have focused on the selectivity and/or affinity towards nuclear GPCRs. Some ligands produced to activate plasma membrane GPCRs have been tested regarding its capability to interact with GPCRs localized in the nuclear membrane. Examples of that are Ang II and Losartan, an agonist and antagonist, respectively, that have been used with success to modulate nuclear angiotensin type 1 (AT_1_) receptors localized in the nuclear membrane of dopaminergic neurons [45], as well as in the kidney of normotensive and hypertensive Lewis rats [46] Interestingly, some AT_1_ receptor antagonists such as Candesartan seem to have an effect mostly on the AT_1_ receptors localized in the plasma membrane while other antagonists such Losartan have shown to block plasma membrane AT_1_ receptors as well as nuclear AT_1_ receptors [47]. Such behavior demonstrates that AT_1_ receptor antagonists seem to present differential selectivity between plasma and nuclear membrane receptors. Isoproterenol is another ligand that has been described to bind to a receptor localized in the nuclear membrane, the nuclear β_3_ adrenoceptor in rat cardiomyocytes [48]. Other nuclear GPCRs, such as nuclear urotensin-II [49] and nuclear B2 [50] receptors, have also been blocked by antagonists that were developed to target their respective plasma membrane counterparts. Despite this evidence, an extended and detailed study is still needed to characterize and clarify the selectivity and affinity of all currently known drugs that use GPCRs as targets towards nuclear GPCRs. Such characterization would allow the identification of drugs/ligands capable of discriminating nuclear GPCRs towards their respective plasma membrane counterparts and, therefore, be more target-directed.

Recent findings regarding the critical influence of lipid composition/structure in the conformational states of GPCRs are also valuable information for the rational design of molecules with the view of obtaining selective ligands for nuclear GPCRs. Medicinal chemistry and the design of new molecules with removable functional groups that allow a higher cell-permeability [51] directing the molecular functional core to the nucleus is another possible strategy to obtain selective nuclear GPCR ligands. These strategies, despite presenting the advantage of delivering the ligand into an intracellular localization and, therefore, turning it to be capable of activating nuclear GPCRs, cannot rule out the putative concomitant activation of respective plasma membrane GPCRs counterparts.

Another possible approach to obtain ligands with an improved selectivity towards nuclear GPCRs relies on the rational design of ligands with allosteric sites, but the application of allosteric binding can be a problem for intracellular sites (reducing ligand specificity as previously described [52]). To overcome these difficulties, the ligand (agonist or antagonist) needs to be designed in a way that ensures a higher affinity and increased binding capabilities for nuclear GPCRs among with the requirement of being able to cross the plasma membrane entering the cell and be diffused into the cytosol, and then, traffic to the nucleus [51]. To address this, ligands (new or already on the market) could be incorporated into nanoparticles [53] or peptides [54,55] which would allow its uptake into cells by taking advantage of cell biological processes (endocytosis and/or exchanger and/or transporters) allowing the putative interaction between the ligand and the targeted nuclear GPCR. As so, a strategy to ensure the target selectivity for nuclear GPCRs may rely, for example, on the use of ligands inside a carrier that is sensitive to intracellular enzymes localized near the nucleus, allowing the delivery of the ligands as pro-drugs into an intracellular environment nearby the nucleus. Thus, ligands that selectively target nuclear GPCRs will constitute valuable tools for researchers to further investigate the mechanisms triggered by nuclear GPCRs and of its ultimate effects. Further studies are also needed to improve or create tools/methods that could enhance the knowledge regarding nuclear GPCRs as targets incrementing the development of ligands that present selectivity for these receptors.

## 4. Nuclear GPCRs in Non-Communicable Diseases

Lipid composition and/or the structure of cell membranes are known to play crucial roles in several cell trafficking and signaling pathways, participating also in the maintenance of homeostasis. As so, lipid composition and/or cell membrane structural changes or lipid imbalance are crucial aspects that can influence the pathophysiological processes that, subsequently, lead to disease [56,57], and may interfere, at least in part, with the way nuclear GPCRs are expressed and function. Such types of phenomena have also been described to occur with other proteins, namely with sphingomyelin and with functional proteins of the nuclear lipid microdomains in cancer cells that increased shuttling of protein signaling molecules [58]. It is also important to highlight the crucial role of lipid composition and/or the structure of cell membranes in the conformational state of GPCRs: any alteration in receptor conformation may modify the receptor binding “pocket” altering the way GPCRs are expected to function. In accordance, it has been shown that negatively charged lipids can stabilize the active state of receptors such as β_2_-adrenoceptors, enabling the docking of G_αs_ protein while neutral zwitterionic lipids seem to inactivate the receptor [59]. Therefore, the GPCRs localized in the nuclear membrane may also be influenced by these phenomena and be involved in the pathophysiology and development of non-communicable diseases [60,61]. Moreover, scientific data also indicate the occurrence of constitutively active mutations in GPCRs that have been associated with diseases [62,63], which can also have a role in the pathophysiological processes.

Regardless of these aspects, studies conducted in cell lines as well as in animal models of diseases appointed for the occurrence of an abnormal expression and/or function of some nuclear GPCRs in pathological conditions associated with non-communicable diseases (Table 1). A redistribution of GPCRs within the cell can occur in healthy state and disease: for instance, in standard metabolic conditions, nuclear parathyroid hormone receptor type 1 (PTH1) and plasma membrane PTH1 revealed a similar expression while in metabolic deprivation conditions, an increase in the nuclear localization of this receptor type occurred contrasting to the reported reduction of plasma membrane PTH1; other examples of GPCRs redistribution (from the plasma membrane to nuclei and vice versa) are shown in Table 2. Interestingly, it has been observed that changes in GPCRs expression, namely the redistribution of receptors from the plasma membrane to nuclear membrane can lead to differential functions of the receptor (Table 2). Moreover, depending on the pathological condition considered, alterations in nuclear GPCRs expressions have also been reported with the occurrence of an upregulation of some type of nuclear GPCRs while in other conditions a downregulation has been reported. Accordingly, the changes in nuclear GPCRs expression depend on the type of pathology as depicted in Table 1. Furthermore, some reports have demonstrated that in specific pathologies, some nuclear GPCRs transduction pathways are deregulated or disruptive, evidencing alterations in the expression and function of the receptor itself and/or of respective signaling mediators. In addition, reports also demonstrated that in diseases such as cardiovascular diseases and cancer some nuclear GPCRs when activated can trigger atypical signaling mediators, therefore, triggering pathways that are not commonly associated with that particular GPCR [64].

As so, targeting such nuclear GPCRs may constitute promising and innovative approaches to treat/prevent these diseases. In the following sections, a summary of the most recent findings regarding the expression and function of GPCRs localized in the nuclear membrane in cancer, neurological and neurodegenerative diseases and cardiovascular diseases will be described.

### 4.1. Nuclear GPCRs in Cancer

In several cancer types, an upregulation of nuclear GPCRs has been observed as shown for the nuclear vasoactive intestinal peptide type 1 (VPAC_1_) receptor (gliomas [82]; breast cancer [77]) and for the nuclear C-X-C chemokine type 4 (CXCR4) receptor (non-small-cell lung cancer [66]). Nuclear VPAC_1_ receptors upregulation has been proposed to be involved in a mechanism of tumor resistance [82] while nuclear CXCR4 receptors expression has been associated with cancer prognosis. Nuclear CXCR4 receptors were associated with a better outcome in an early-disease stage in non-small-cell lung cancer [66] while for nasopharyngeal carcinoma [72], renal carcinoma [85,86,97], colorectal cancer [73] and gastric cancer [84], the nuclear expression of CXCR4 receptors seems to be linked with poor survival and an increased metastatic capacity. In fact, in nasopharyngeal carcinoma, nuclear CXCR4 receptors could be used as a prognostic factor since the association between cancer progression and poor overall survival has been previously reported [64]. Likewise, regarding colorectal cancer, nuclear but not cytoplasmic expression of this receptor was associated with advanced cancer and lymphovascular invasion [73]. For renal carcinoma, nuclear CXCR4 receptors persistently demonstrated to be a promoter of metastasis [78,85,86] and the receptor’s localization at the nuclear membrane was only found in metastatic renal cell carcinoma lesions [85].

**Figure 2:**
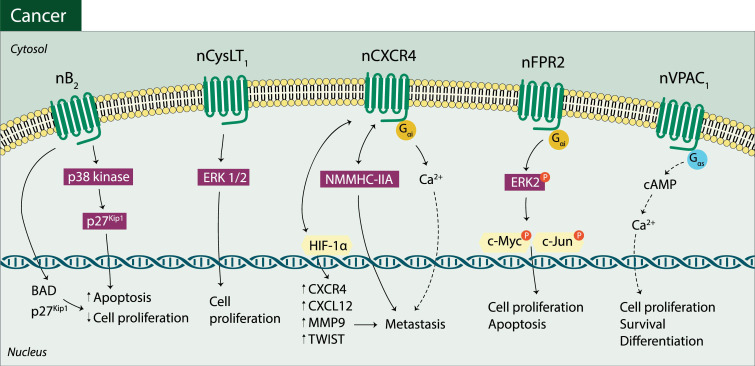
Atypical pathways involving nuclear GPCRs in cancer: nuclear bradykinin B type 2 (nB_2_), nuclear cysteinyl leukotriene type 1 (nCysLT_1_), nuclear C-X-C chemokine type 4 (nCXCR4), nuclear N-formyl peptide type 2 (nFPR) and nuclear vasoactive intestinal peptide type 1 (nVPAC_1_) receptor activated signaling pathways.

Changes in the signaling pathways triggered by nuclear GPCRs have been identified in cancer (Table 1) with the occurrence of increased levels of signaling mediators and of protein phosphorylation. An atypical activity ascribed to nuclear GPCRs activation has been reported: activation of some nuclear GPCRs has been shown to trigger different signaling events and pathways relatively to those commonly described for the respective plasma membrane GPCRs. As such, these GPCRs and respective pathways can be viewed as putative targets presenting a promising role in cancer therapy (Figure 2).

**Table 2:** Differential expression between nuclear and plasma membrane GPCRs in physiological and pathophysiological conditions.

Receptor	Nuclear Membrane	Plasma Membrane	Cell Type/*Function*	Ref.
mGlu_5_	+	++	Striatal neurons	[98]
			*growth/differentiation*	
mGlu_5_	++	++	Striatal neurons	[98]
			*synaptic plasticity and growth/differentiation*	
mGlu_5_	+	++	spinal dorsal horn neurons	[94]
			*without pain*	
mGlu_5_	+++	++	spinal dorsal horn neurons	[94]
			*persistent pain*	
S1P1		++	unstimulated T-cells	[99]
			*>cell migration*	
S1P1	+++	+	Stimulated T-cells	[99]
			*<cell proliferation*	
F2rl1		++	Vascular cells	[93]
			*>vessel maturation*	
F2rl1	+++	++	Vascular cells	[93]
			*>angiogenesis*	
PTH1	++	++	Osteoblasts	[36,100]
			*Standard metabolism*	
PTH1	+++	++	Osteoblasts	[36,100]
			*Metabolic deprivation*	

Abbreviations: F2rl1—coagulation factor II receptor-like 1; mGlu_5_—metabotropic glutamate type 5 receptor; PTH1—parathyroid hormone type 1 receptor; S1P1—sphingosine-1-phosphate receptor. definition amount of receptors: +, low; ++, middle; +++, high.

In accordance with this, the modulation of nuclear B_2_ receptors with cell-permeable antagonists (for this type of receptor) proved their involvement in human triple-negative breast cancer via a mechanism involving p38 mitogen-activated protein kinase (p38 kinase)—cyclin-dependent kinase inhibitor 1B (p27^kip1^) leading to apoptosis and reducing cell proliferation [80]. Thus, nuclear B_2_ receptors may constitute a promising target that when inhibited can reduce cancer progression. Moreover, other GPCR, the N-formyl-peptide type 2 (FPR2) receptor was described to be expressed at a nuclear level in lung carcinoma, triggering intranuclear signaling that leads to enhanced extracellular signal-regulated kinase 1/2 signaling (ERK1/2), c-Jun and c-Myc phosphorylation, and therefore, affecting cell cycle, apoptosis and cell proliferation [39].

Another report described that the orphan receptor, probable G-protein coupled 158 (GPR158) receptor promotes prostatic cell proliferation (in human prostate cancer cells) independently of androgen receptor function and stimulates androgen receptors and prostate specific antigen expression. Simultaneously, an alternative mechanism, consistent with the localization of these receptors in the nuclei of cells, promoting cell proliferation is activated [71], thus the GPR158 receptors blockade may reduce cell proliferation and cancer development. Moreover, in cancer, namely in colorectal adenocarcinomas, activation of nuclear cysteinyl leukotriene type 1 (CysLT_1_) receptor seems to trigger proliferative ERK1/2 contributing to inflammation-induced colon carcinogenesis [38]. As so, inhibition of nuclear CysLT_1_ receptors can also constitute a putative strategy to reduce carcinogenesis and lead to a better treatment outcome in colon carcinoma.

### 4.2. Neurological and Neurodegenerative Diseases

**Figure 3:**
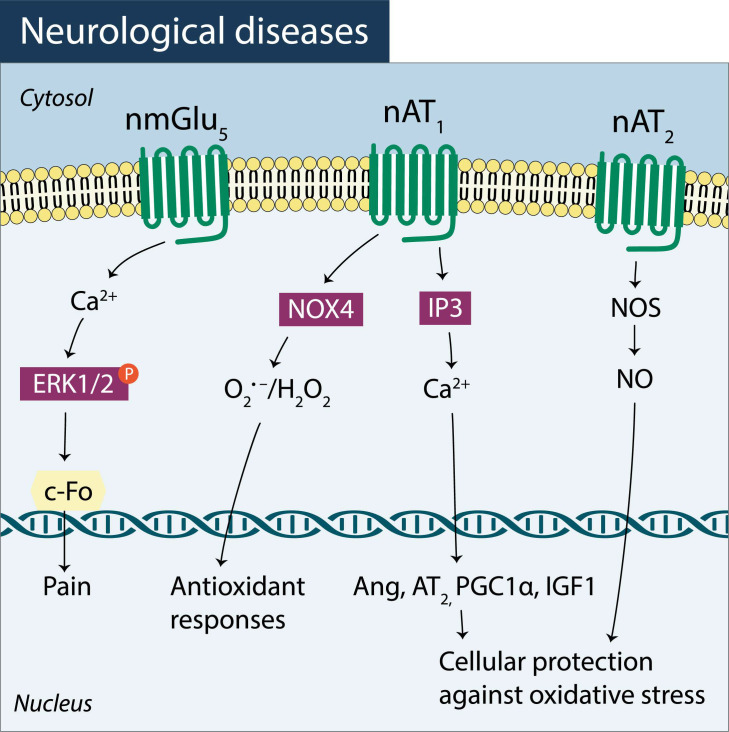
Atypical pathways involving nuclear GPCRs in neurological and neurodegenerative diseases: nuclear metabotropic glutamate type 5 (nmGLU_5_), nuclear angiotensin type 1 (nAT_1_) and type 2 (nAT_2_) receptor activated signaling pathways.

Evidence also demonstrates the occurrence of nuclear GPCRs-mediated mechanisms in neurological and neurodegenerative diseases (Figure 3). High levels of Ang II were reported to increase oxidative stress and to promote neuroinflammation [101] and, in dopaminergic neurons, had led to an overexpression of nuclear AT_1_ receptors while the expression of nuclear angiotensin type 2 (AT_2_) receptors was downregulated [45].

Activation of nuclear AT_1_ receptors by Ang II has been described to trigger protective mechanisms involving, for instance, increase levels of peroxisome proliferator-activated receptor gamma coactivator 1-alpha (PGC-1α) and insulin-like growth factor 1 (IGF-1)—sirtuin 1 (SIRT1) [45] modifying mitochondria biogenesis/function. Since the nervous system depends on ATP levels for the maintenance of ionic gradients across the cell membranes and for neurotransmission, improvement of mitochondria biogenesis has a substantial beneficial impact on neuronal function and, thus explains why nuclear AT_1_ receptor can be protective in nervous system diseases.

Another effect of nuclear GPCRs in the nervous system has also been described in spinal dorsal neurons where an upregulation of nuclear metabotropic glutamate type 5 (mGlu_5_) receptors occurred resulting in ERK1/2 activation as well as into an activity-regulated cytoskeleton-associated protein (Arc/Arg3.1) activation, causing an upregulation of c-fos expression [95] and, thus, evidencing a critical role for nuclear mGlu_5_ receptors in neuropathic pain. In the literature, it has been described that in persistent pain, in spinal dorsal horn neurons, most of mGlu_5_ receptors are localized intracellularly in the nucleus where they are functionally active [94].

Furthermore, it has been observed that during aging the nuclear distribution and responses of GPCRs can also present considerable changes since evidence indicates a decrease in nuclear AT_1_ and nuclear AT_2_ receptors in aged brains, accompanied by an impairment of several compensatory mechanisms contributing to the neurodegenerative processes associated with aging [45].

All the nuclear GPCRs above described constitute, therefore, putative therapeutic targets that need to be further studied in neurological and neurodegenerative diseases.

### 4.3. Cardiovascular Diseases

In several cardiovascular diseases, modifications in the expression of the GPCRs localized in the nuclei membrane were also described [29]. For example, an upregulation was found in nuclear AT_1_ receptors in heart failure [31] and in fetal programming of hypertension induced by glucocorticoids [90] while, by opposition, a downregulation of nuclear AT_1_ receptors was observed in hypertension [89]. Furthermore, the nuclear AT_2_ receptors expression in fetal programming of hypertension [90] has been demonstrated to be downregulated.

**Figure 4:**
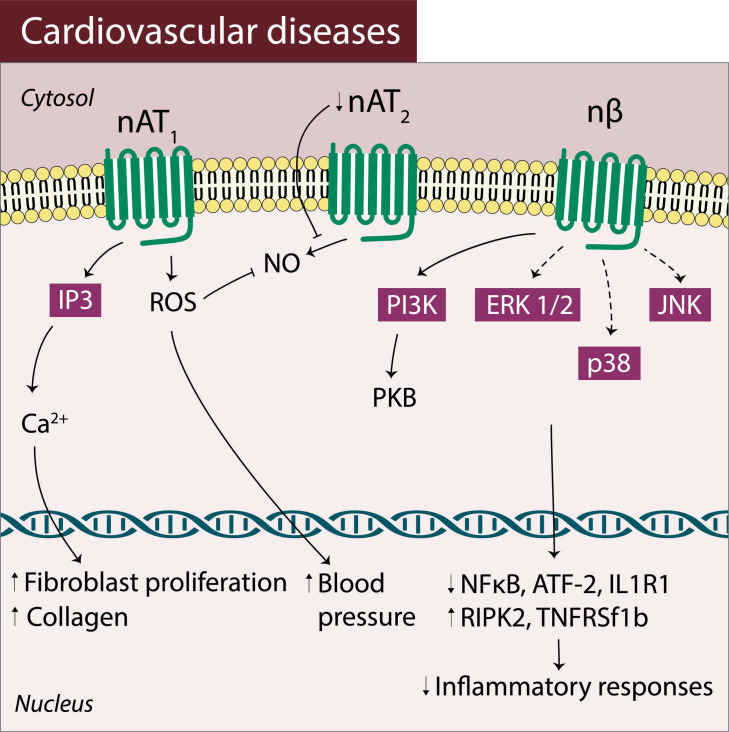
Atypical pathways involving nuclear GPCRs in cardiovascular diseases: nAT_1_ and nAT_2_ receptor and nuclear β-adrenoceptor (nβ) activated signaling pathways in cardiovascular diseases.

In addition, an altered activity exerted by nuclear GPCRs has also been described in cells from the cardiovascular system contributing to disease (Figure 4). For instance, in heart failure, nuclear AT_1_ receptors have led to an increase in nuclear Ca^2+^ having an impact both in the subsequent regulation of fibroblast proliferation and in the collagen gene expression/secretion [31]. Moreover, in fetal programming of hypertension, nuclear AT_1_ receptors have been described to be activated and to mediate an increase in ROS generation as well as a decrease of NO [46,90]. Both effects involve the AT_1_ receptors localized in the nuclear membrane and seem to contribute to an increase in blood pressure.

Moreover, nuclear GPCRs atypical activity has also been described associated with the nuclear β—adrenoceptors (nuclear β_1_ and/or nuclear β_3_ subtypes) that may also interact with endothelin (ET-1), modulating transcription factors generation (Figure 4). Activation of nuclear β_1_ and/or nuclear β_3_ adrenoceptors seems to cause a reduction of nuclear factor kappa-light-chain-enhancer of activated B-cell (NF-κB), an activation of transcription factor 2 (ATF-2), of the interleukin-1 type 1 (IL1r1) receptor and an increase of tumor necrosis factor receptor superfamily member 1B (Tnfrsf1b) transcription in inflammation related with congestive heart failure and atrial fibrillation [88].

These GPCRs may constitute putative targets that should be considered for some (already identified) cardiovascular diseases.

## 5. GPCR-Based Drugs in the Treatment of Non-Communicable Diseases: Implication of Nuclear GPCRs as Targets

Most drugs currently in use in clinical practice to treat and/or prevent several types of diseases namely non-communicable diseases, are GPCR-based drugs. The occurrence of nuclear GPCRs as new targets is supported by recent data that has identified several atypical mechanisms and altered signaling pathways triggered by GPCRs localized in the nuclear membrane (Table 1 and Figure 2, Figure 3 and Figure 4). These findings have also highlighted the occurrence of differences in the function of nuclear GPCRs towards plasma membrane GPCRs.

It is conceivable that some of the clinical effects attributed so far to the GPCR-based drugs already in the market (that have been considered to act solely on the plasma membrane GPCRs) can be ascribed, at least in part, to the activation or blockage of GPCRs localized in the nuclear membrane. For example, AT_1_ receptor antagonists such as Losartan, in use in the clinical practice to treat hypertension, can promote vasodilation of smooth muscle cells (diminishing blood pressure), an effect that has been attributed to plasma membrane AT_1_ receptors blockade, but in agreement with data recent described may also address an additional mechanism of action involving the AT_1_ receptors localized in the nuclear membrane [31,90]: nuclear AT_1_ receptors when blocked reduced oxidative stress and Ca^2+^ generation, both effects also influencing vasodilation.

The same rationale can be applied to other GPCR-based drugs used in clinical practice to treat non-communicable diseases. For instance, Isoprenaline or Salmeterol, both β-adrenoceptor agonists, in use to treat arrhythmias and asthma, respectively. Another example is Montelukast, a CysLT_1_-selective antagonist, in use to treat asthma. These drugs act on GPCRs that have been reported in the plasma membrane but that recently have also been identified in the nuclear membrane and, these later receptors (nuclear GPCRs) may address, at least in part, their clinical efficacy (if we consider that nuclear GPCRs may reinforce the effects mediated by plasma membrane GPCRs).

In this sense, the occurrence in the nuclear membrane of receptors belonging to the renin angiotensin system (RAS), such as nuclear AT_1_ and nuclear AT_2_ receptors, already identified in cells from cardiovascular and nervous systems (see Table 1), highlight the possibility that these receptors may also be involved in the regulation of biological processes mediated by other players of RAS: it has been demonstrated that increased activation of plasma membrane AT_1_ receptors by Ang II triggers ERK1/2 and p38 MAPK signaling pathways, which causes a downregulation of angiotensin converting enzyme type 2 (ACE2) expression and, by opposition, an upregulation of angiotensin converting enzyme type 1 (ACE) [102]. Taking this into account, the eventuality that AT_1_ or AT_2_ localized in the nuclear membrane may also participate in the regulation of ACE and of ACE2 expressions should be put forward. It is also conceivable that effects mediated by nuclear GPCRs could block the initial responses triggered by plasma membrane GPCRs activation. In both cases, nuclear GPCRs would have a major impact on the pharmacological outcome and, consequently, in the therapy.

Since redistribution of GPCRs has been reported (Table 2) and can occur in healthy conditions as well as in diseases (and may differ depending on the progression of the disease), a GPCR ligand may exert effects that depend on the level of expression and/or distribution profile of GPCRs (as targets) localized in the nuclear versus plasma membrane. As so, the activation of the nuclear GPCRs may trigger responses conditioning the therapeutic outcome. In fact, nuclear receptors mediated actions can be distinct from those elicited by its respective plasma membrane counterparts. Furthermore, it is also plausible that some of the nuclear GPCR-mediated mechanisms might explain some side effects or unusual effects ascribed to some drugs in the clinical practice (for examples please see [27]).

Despite most of the nuclear GPCRs and plasma membrane GPCRs have identical structures, some exceptions have been reported: in frizzled-2 (FZD2) receptor, only a small functional portion of the heptahelical chain, without the C-terminus, seem to be translocated into the nuclear membrane [103]; GPCRs might undergo post-translational modifications that differ between the plasma and nuclear membranes [49], as occurs for the endothelin type B (ET_B_) receptor since only the plasma membrane receptor undergoes N-glycosylation, while in nuclear ET_B_, receptor N-glycosylation is absent [104]. Another major structural feature that has an impact on GPCRs responses relies on plasma membrane lipid composition and/or structure and their respective influence on GPCR conformation [61,105,106]. To our knowledge, at present, there is no information in the literature regarding the influence of lipid composition on nuclear GPCRs dynamics, but it has been observed that lipid partitioning controls membrane biogenesis at the nuclear envelope [107] suggesting that nuclear lipid composition or structure can be modified. In addition, some studies have indicated that lipid composition influences nuclear receptors’ function such as androgen receptors, glucocorticoid receptors, among others [105,108]. Additionally, the occurrence of nuclear microdomains lipid composition has been described to change according to cell status, being increased, for instance, in cell proliferation [109,110]. Thus, altogether these features appoint for the possibility that altered nuclear GPCR activities result, at least in part, from modified lipid composition and structure of the nuclear membrane, which would influence the access of ligands to nuclear GPCRs but also modify nuclear GPCR self-responses to ligands, thus conditioning the therapeutic outcomes. This possibility needs to be explored in future studies to provide new insights on the mechanisms of nuclear GPCR altered functions and/or expressions.

## 6. Conclusions

In recent years nuclear GPCRs have evidenced important physiological roles, namely in the regulation of cell proliferation, transcription, apoptosis, angiogenesis and cell survival. In multiple pathological conditions such as non-communicable diseases (cancer, neurological and neurodegenerative diseases as well as cardiovascular diseases), changes in nuclear GPCRs have been described, namely with the occurrence of modified expressions and/or of atypical and/or disruptive signaling pathways (triggered by nuclear GPCRs) which highlight nuclear GPCRs as promising therapeutic targets. Future studies are required to better understand the new intracellular roles of GPCRs including the selectivity and/or affinity of tailor ligands for both nuclear and plasma membrane GPCRs, contributing, therefore, to improve GPCR-based therapeutics to non-communicable diseases.

## Figures and Tables

**Table 1:** Nuclear GPCRs occurrence in non-communicable diseases.

Pathology	Type/Sample	Model	Nuclear GPCR	Effectors/Mediators and/or Effects	Ref
Cancer	Lung	Human non-small-cell lung cancer	CXCR4		[65]
		Human non-small-cell lung cancer tissue	↑CXCR4	associated with a better outcome	[66]
		Human primary non-small cell lung cancer tissue	CXCR4	aberrant nuclear CXCR4 expression ↔ lymph node metastasis	[67]
		Human lung carcinoma cell line	FPR2	Gαi—ERK2, c-Jun and c-Myc phosphorylation	[35]
	Liver	Human hepatoma cancer cells	CXCR4	─	[68]
		Transfected HTC4 rat hepatoma cells	LPA1	─	[69]
	Prostate	Human prostate cancer cell lines	↑CXCR4	Gαi—↑Ca2+; ↑ nuclear CXCR4 with tumor grade	[70]
		Human prostate cancer cell lines	GPR158	promotes cell proliferation	[71]
	Oral	Nasopharyngeal carcinoma	CXCR4	associated with the cancer progression	[72]
	Colon	Human colon adenocarcinoma cell line	VPAC		[30]
	Colorectal	Human colorectal cancer tissue	↑CXCR4	associated with poor overall survival	[73]
			↑CXCR4	nuclear CXCR4—more frequent lymph node metastasis	[74]
			↑CysLT_1_	proliferative ERK1/2 signaling	[38]
	Placenta	Human placental choriocarcinoma cell lines	MT2	─	[75]
	Bone	Human osteosarcoma (U2OS, MG63, OS15 and SaOS2)	OT	─	[76]
	Breast	Human breast carcinoma cell lines (T47D, MDAMB-468)	↑VPAC_1_	Gαs- ↑ cAMP	[77]
		Human breast cancer (MCF7)	OT	─	[76]
		Human ductal carcinoma tissue	CXCR4	─	[78]
		Human triple-negative breast cancer	↑B_1_	cell-permeable antagonists have superior antineoplastic activity	[79]
			↑B_2_	cell-permeable antagonists have superior antineoplastic activity; anti-proliferative effects through p38kinase/p27Kip1	[80]
	Brain	Human glioblastoma-astrocytoma U87-MG and human neuroblastoma SH-SY5Y cell lines	UT	transcription initiation	[49]
		Glioblastoma multiforme cell lines	CXCR4	─	[81]
		Glioblastoma multiforme cell lines	↑VPAC_1_VPAC_2_	↑nuclear VPAC1 with glioma grade	[82]
	Gastric	Human gastric adenocarcinoma tissue and cell line	CXCR4	nuclear CXCR4 expression ↔ better overall survival	[83]
		Primary gastric cancer tissue	CXCR4	nuclear CXCR4 expression ↔ reduced survival rate	[84]
		Human gastric adenocarcinoma cell line	FPR2	Gαi—ERK2, c-Jun and c-Myc phosphorylation	[35]
	Renal	Human renal carcinoma cell lines	CXCR4	in the nucleus only in metastatic lesions	[85]
			CXCR4	interaction and nuclear accumulation of HIF-1α—metastasis promotion	[86]
			CXCR4	interaction with myosin heavy chain-IIA—CXCR4 nuclear translocation—↑tumor metastatic capacity	[78]
		Human renal cancer tissue	CXCR4	associated with metastasis and poor survival	[87]
Cardiovascular Diseases	Inflammation	Rat cardiomyocytes	β	Gαi—PI3K—PKB—ERK1/2 -↓NF-κB transcription—↓ATF-2, IL1r1 and Tnfrsf1b + ↑Ripk2 transcription → suppression of inflammatory response	[88]
	Hypertension	Hypertension model—rat kidney	↓AT_1_	─	[89]
		Fetal programming model—sheep kidney	↑AT_1_, ↓AT_2_	↑ROS, ↓NO	[90]
	Heart failure	Heart failure model—canine cardiac fibroblasts	↑AT_1_	AT1—IP3—↑Ca2+—regulate fibroblast proliferation, collagen gene expression and collagen secretion	[31]
	Angiogenesis	Human umbilical vein endothelial cells	S1P_1_	Cyr61 and CTGF expression	[91]
		Model of oxygen-induced retinopathy—rat ocular tissue	PAF	VEGF-dependent neovascularization in oxygen-induced retinopathy	[92]
		Mouse retinal ganglion cells	F2rl1	Sp1 recruitment—↑VEGFα expression → neovascularization	[93]
Neurological and neurodegenerative diseases	Neuropathic pain	Rat spinal dorsal horn neurons	↑mGlu_5_	Nerve injury—↑nuclear mGlu5—↑[Ca2+]n + ERK1/2 and Arc/Arg3.1 activation + ↑ c-fos expression	[94]
	Nociceptive	Rat spinal dorsal horn neurons	↑mGlu_5_	Inflammation—↑nuclear mGlu5—↑ c-fos expression	[95]
	Oxidative stress	Rat and dopaminergic neurons cell line	AT_1_, AT_2_	AT1—IP3—↑Ca2+ → ↑AT2 + Ang + PGC-1α + IGF-1 transcription → cellular protection; AT1—NOX4—↑superoxide/H2O2 → antioxidant response; AT2—NOS—↑NO	[45]
	Epilepsy	Epilepsy model—rat hippocampus	B_1_, B_2_	changes in receptors’ distribution during acute, silent and/or chronic periods	[96]

Abbreviations: Ang—angiotensin; Arc/Arg3.1—activity-regulated cytoskeleton-associated protein; ATF-2—activating transcription factor 2; AT_1_—angiotensin receptor type 1; AT_2_—angiotensin receptor type 2; B_1_—bradykinin receptor type 1; B_2_—bradykinin receptor type 2; Ca^2+^- calcium; cAMP—cyclic adenosine monophosphate; CTGF—connective tissue growth factor; CXCR4—C-X-C chemokine receptor type 4; Cyr61—cysteine-rich angiogenic protein 61; CysLT_1_—cysteinyl leukotriene receptor type 1; ERK2—extracellular signal-regulated kinase 2; ERK1/2—extracellular signal-regulated kinase 1/2; FPR2—N-formyl peptide receptor type 2; F2rl1—coagulation factor II receptor-like 1; GPR158—G-protein coupled receptor 158; HIF-1α—hypoxia-inducible factor 1-α; H2O2—hydrogen peroxide; IGF-1—insulin-like growth factor-1; IL1r1—interleukin-1 receptor type 1; IP3—inositol trisphosphate; JNK—c-Jun N-terminal kinase; LPA1—lysophosphatidic acid receptor type 1; mGlu_5_—metabotropic glutamate receptor type 5; MT2—melatonin receptor type 2; NF-κB—nuclear factor kappa-light-chain-enhancer of activated B-cell; NO—nitric oxide; NOX4—nicotinamide adenine dinucleotide phosphate (NADPH) oxidase 4; OT—oxytocin receptor; PAF—platelet-activating factor receptor; PGC-1α—peroxisome proliferator-activated receptor gamma coactivator 1; αPI3K—phosphoinositide 3-kinase; PKB—protein kinase B; p27/kip1—cyclin-dependent kinase inhibitor 1B; p38kinase—p38 mitogen-activated protein kinase; Ripk2—receptor-interacting serine/threonine-protein kinase 2; ROS—reactive oxygen species; S1P1—sphingosine-1-phosphate receptor; Tnfrsf1b—tumor necrosis factor receptor superfamily member 1B; UT—urotensin-II receptor; VEGF—vascular endothelial growth factor; VPAC—vasoactive intestinal peptide receptor; VPAC_1_—vasoactive intestinal peptide type 1 receptor; VPAC_2_—vasoactive intestinal peptide type 2 receptor.
